# From risk to chronicity: genetic and neuroimaging insights into the evolving patterns of spontaneous brain activity in schizophrenia

**DOI:** 10.1017/S0033291725102006

**Published:** 2025-10-10

**Authors:** Yijing Zhang, He Wang, Mengjing Cai, Wei Wang, Jin Qiao, Xinyu Wang, Yue Wu, Qian Wu, Zhihui Zhang, Minghuan Lei, Qi An, Wenjie Cai, Haolin Wang, Fengtan Li, Yingying Xie, Feng Liu, Lining Guo

**Affiliations:** 1 Department of Radiology and Tianjin Key Laboratory of Functional Imaging & Tianjin Institute of Radiology, Tianjin Medical University General Hospital, Tianjin, China; 2 Department of Medical Imaging, Henan Provincial People’s Hospital, Zhengzhou, China; 3 Department of Radiology, The First Affiliated Hospital of Fujian Medical University, Fuzhou, China

**Keywords:** gene expression, resting state fMRI, schizophrenia, spontaneous brain activity, transcriptome-neuroimaging association analysis

## Abstract

**Background:**

Schizophrenia progresses through high-risk, first-episode, and chronic stages, each associated with altered spontaneous brain activity. Resting state functional MRI studies highlight these changes, but inconsistencies persist, and the genetic basis remains unclear.

**Methods:**

A neuroimaging meta-analysis was conducted to assess spontaneous brain activity alterations in each schizophrenia stage. The largest available genome-wide association study (GWAS) summary statistics for schizophrenia (*N* = 53,386 cases, 77,258 controls) were used, followed by Hi-C-coupled multimarker analysis of genomic annotation (H-MAGMA) to identify schizophrenia-associated genes. Transcriptome-neuroimaging association and gene prioritization analyses were performed to identify genes consistently linked to brain activity alterations. Biological relevance was explored by functional enrichment.

**Results:**

Fifty-two studies met the inclusion criteria, covering the high-risk (*N*_high-risk_ = 409, *N*_control_ = 475), first-episode (*N*_case_ = 1842, *N*_control_ = 1735), and chronic (*N*_case_ = 1242, *N*_control_ = 1300) stages. High-risk stage showed reduced brain activity in the right median cingulate and paracingulate gyri. First-episode stage revealed increased activity in the right putamen and decreased activity in the left gyrus rectus and right postcentral gyrus. Chronic stage showed heightened activity in the right inferior frontal gyrus and reduced activity in the superior occipital gyrus and right postcentral gyrus. Across all stages, 199 genes were consistently linked to brain activity changes, involved in biological processes such as nervous system development, synaptic transmission, and synaptic plasticity.

**Conclusions:**

Brain activity alterations across schizophrenia stages and genes consistently associated with these changes highlight their potential as universal biomarkers and therapeutic targets for schizophrenia.

## Introduction

Schizophrenia is a complex mental disorder characterized by profound disturbances in thought processes, cognition, and behavior, expressed through recurring episodes of psychosis (McCutcheon, Reis Marques, & Howes, 2020). The progression of schizophrenia through various stages is marked by a spectrum of clinical manifestations, with each stage providing unique insights into the evolving nature of the disorder. At the high-risk stage, individuals often exhibit subtle cognitive and behavioral anomalies, serving as early indicators of potential psychosis (Mayeli, Clancy, Sonnenschein, Sarpal, & Ferrarelli, 2022; Mittal et al., 2010; Montag et al., 2020). Upon transitioning to the first-episode phase, individuals experience overt psychotic symptoms such as hallucinations, delusions, and disorganized thinking (Fitzsimmons et al., 2014; Kahn et al., 2015; Melvin, Crossley, & Cromby, 2021). As the illness progresses into the chronic phase, a myriad of cognitive, affective, and social impairments becomes more entrenched (Kahn & Keefe, 2013; Ortiz-Gil et al., 2018; Siever & Davis, 2004). Individuals in this stage often grapple with persistent and disabling symptoms, impacting their daily functioning and quality of life (Mathalon, 2011; Uno & Coyle, 2019). By concurrently examining these stages, researchers can delineate the continuum of the disorder, enabling a detailed examination of how abnormalities evolve over time and how interventions can be adapted to address specific needs at different points in the illness. As schizophrenia advances through its stages, understanding the underlying neurobiological alterations becomes crucial for gaining a comprehensive insight into the disorder.

Neuroimaging techniques offer a unique perspective on the dynamic changes within the brain across different phases in schizophrenia, providing valuable insights into the neural underpinnings of cognitive and behavioral disturbances observed in high-risk, first-episode, and chronic stages. One such noninvasive method is resting state functional magnetic resonance imaging (rs-fMRI), capturing spontaneous fluctuations in blood oxygen level-dependent (BOLD) signals during rest (Hoge, 2012). Existing studies have identified both common and distinct changes in spontaneous brain activity across various stages of schizophrenia, with characteristic alterations emerging at the high-risk, first-episode, and chronic stages of the disorder (Fryer et al., 2016; Li et al., 2016; Ma, Yang et al., 2023; Ren et al., 2013; Yu et al., 2014). Analyzing spontaneous brain activity mainly involves several approaches, including regional homogeneity (ReHo), amplitude of low-frequency fluctuations (ALFF), and fractional amplitude of low-frequency fluctuations (fALFF) (Gao et al., 2023; Hannawi, Lindquist, Caffo, Sair, & Stevens, 2015; Li et al., 2023; Meier et al., 2020; Qiu et al., 2019). ReHo evaluates the synchronization of BOLD signal time series within a local cluster (Zang, Jiang, Lu, He, & Tian, 2004), while ALFF quantifies the amplitude of low-frequency oscillations in the BOLD signal. fALFF, a normalized version of ALFF, divides low-frequency amplitude by the total power across all frequencies (Han et al., 2011; Zou et al., 2008).

A critical issue in current research on schizophrenia is the imbalance in stage-specific investigations. Most studies have predominantly focused on discrete stages – particularly the first-episode and chronic phases – while largely overlooking the high-risk period and the longitudinal transitions between stages. This lack of a continuous, developmental perspective has hindered a comprehensive understanding of the neurobiological progression of the illness. Specifically, it limits the identification of early biomarkers, obscures the mapping of dynamic trajectories, and constrains efforts to define optimal windows for stage-specific prevention and intervention. Additionally, studies face challenges with inconsistent findings on altered spontaneous brain activity at each stage (Fryer et al., 2016; Huang et al., 2022), likely due to factors such as small sample sizes, variations in participants’ demographic and clinical characteristics, and differences in analytic protocols. In light of these challenges, leveraging neuroimaging based meta-analyses becomes crucial for aggregating and synthesizing findings across diverse studies (Cai et al., 2023; Guo et al., 2023; Ma, Xue et al., 2023; Zhang et al., 2025). This approach not only accommodates the variability in individual study results, but also facilitates a more comprehensive understanding of the collective impact of spontaneous brain activity alterations in schizophrenia across its entire spectrum. Furthermore, neuroimaging meta-analysis can be used to compare brain activity alterations across distinct stages of schizophrenia, allowing for the identification of commonalities and differences in neural signatures across stages, shedding light on potential biomarkers or therapeutic targets specific to each phase (Zhao, Lau et al., 2022; Zhao, Zhang et al., 2022).

Despite the valuable insights gained from neuroimaging studies into the brain’s dynamic changes across different stages of schizophrenia, the genetic mechanisms underlying these brain activity alterations remain underexplored. Schizophrenia is highly heritable, with estimates from twin studies suggesting a heritability of around 80% (Hilker et al., 2018; McCutcheon, Reis Marques et al., 2020). Genetic factors are pivotal in the etiology of schizophrenia, shaping both susceptibility to the disorder and its manifestations across various stages (Owen, Legge, Rees, Walters, & O’Donovan, 2023; Pergola, Penzel, Sportelli, & Bertolino, 2023). Investigating the shared genetic influences that persist through different stages of schizophrenia can reveal core biological pathways underlying its fundamental pathology, beyond stage-specific symptoms. Genome-wide association studies (GWAS) have been essential in identifying specific genetic variations associated with schizophrenia, revealing numerous risk loci and highlighting the polygenic nature of the disorder (Li et al., 2017; Trubetskoy et al., 2022). However, the genetic basis of spontaneous brain activity differences in schizophrenia, which relies on group-level comparisons, remains largely unknown, as the GWAS approach is designed for individual phenotypes and is thus unsuitable for such investigations. The advent of imaging transcriptomics has emerged as a powerful tool to address this gap (Arnatkeviciute, Markello, Fulcher, Misic, & Fornito, 2023; Xue et al., 2022; Xue et al., 2023). Advancements in this field enable the creation of comprehensive brain-wide transcriptional atlases, presenting unparalleled opportunities to thoroughly explore the molecular foundations of neuroimaging phenotypes, whether derived from individual-level analyses or group-level comparisons. Up to the present, imaging transcriptomics has successfully unveiled the molecular correlates of brain activity in various disorders, such as major depressive disorder and autism spectrum disorder (Berto et al., 2022; Sun et al., 2023).

**Figure 1.**
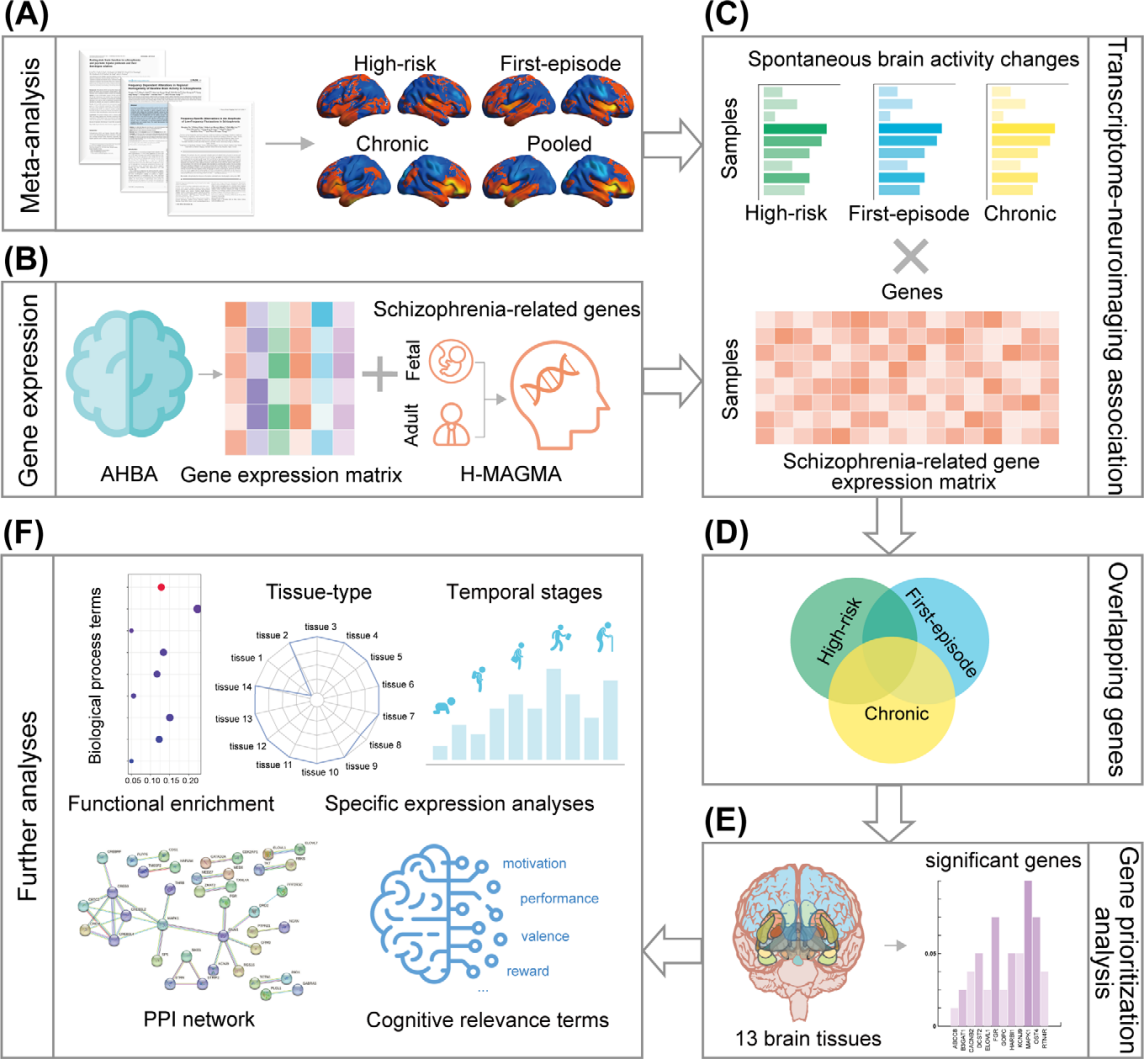
A systematic flowchart of the study design. (a) Neuroimaging meta-analysis was used to identify spontaneous brain activity differences for each stage of schizophrenia as well as across all three stages combined. (b) Schizophrenia-associated genes were identified using H-MAGMA after preprocessing the gene expression data. (c) Transcriptome-neuroimaging association analysis was conducted to identify genes linked to spontaneous brain activity across different stages of schizophrenia. (d) Overlapping genes identified among the three stages. (e) Gene prioritization analysis. (f) Further analyses, including functional enrichment, specific expression, PPI, and behavioral relevance analyses, were performed. AHBA, ‘Allen Human Brain Atlas’; H-MAGMA, ‘Hi-C-coupled multimarker analysis of genomic annotation’; PPI, ‘protein–protein interaction’.

In this study, we conducted a neuroimaging meta-analysis and transcriptome-neuroimaging association analysis to unveil the genetic mechanisms underlying brain activity alterations associated with schizophrenia. Specifically, we performed voxel-wise meta-analyses to examine brain activity changes across different stages of schizophrenia, including high-risk, first-episode, and chronic phases. This approach allowed us to identify common and distinct patterns of brain activity alterations related to the progression of the disorder. Following this, we integrated transcriptomic data by conducting a transcriptome-neuroimaging association analysis, linking gene expression profiles from the Allen Human Brain Atlas (AHBA) with the neuroimaging findings. By focusing on genes consistently associated with brain activity changes across the different stages of schizophrenia, this study aims to identify universal biomarkers and therapeutic targets, enabling earlier diagnosis and treatments that effectively address schizophrenia across its entire course. A schematic overview of our analytical framework is provided in Figure 1.

## Methods

### Search strategy and selection criteria

A comprehensive literature search was conducted in PubMed, Embase, and Web of Science to retrieve studies published before November 2023 using the following search terms: ‘ReHo’ or ‘regional homogeneity’ or ‘ALFF’ or ‘amplitude of low-frequency fluctuation’ or ‘low-frequency fluctuation’ or ‘fALFF’ or ‘fractional amplitude of low-frequency fluctuation’ or ‘fMRI’ or ‘functional magnetic resonance imaging’ and ‘psychosis’ or ‘psychotic’ or ‘schizophrenia’ or ‘schizoaffective’ or ‘schizophrenia disorder’, combined with the keywords ‘resting state’ or ‘rest’. Additionally, the reference lists of included studies and relevant scholarly reviews were searched for any additional studies.

The studies were included if they met all the following criteria: (1) being original, peer-reviewed articles published in English; (2) including participates aged from 18 to 60 years; (3) conducting comparisons of ReHo, ALFF, or fALFF at a whole-brain level between patients with schizophrenia and healthy controls (HCs); (4) providing coordinates of significant clusters in a standardized anatomical space (e.g. MNI space), or reported null findings. Articles were excluded if they: (1) performed voxel-wise comparisons within regions of interest; (2) involved animal experiments; or (3) lacked peak coordinates, even after attempting to obtain this information from the corresponding authors. The high-risk phase in our study was defined as first-degree relatives of schizophrenia patients or individuals with psychosis risk syndrome (Miller et al., 2002; Miller et al., 2003; Yung et al., 2016). The patients with first-episode and chronic schizophrenia were defined as those with an illness duration of less than 2 years and more than 5 years, respectively (Yang et al., 2021; Zhao et al., 2018). Studies with the largest sample sizes were prioritized if the data were obtained from the same resources. If an article reported multiple independent patient samples or distinct neuroimaging metrics, we considered them as separate datasets. Additionally, for studies with a longitudinal design, only the baseline comparison between patients and HCs was considered for inclusion. Our meta-analysis adhered to the guidelines outlined in the Preferred Reporting Items for Systematic Reviews and Meta-Analyses (PRISMA) (Liberati et al., 2009).

The following information was extracted from each included study: demographic characteristics (e.g. sample size, mean age, gender, and education years) and clinical characteristics (e.g. illness duration and medication status), as well as details on data acquisition and processing methods (e.g. MRI scanner type, head coil, slice thickness, and smoothing kernel size). Furthermore, peak coordinates and corresponding statistics (e.g. *t* values) were also documented. The assessment of study quality was carried out using a 10-point checklist, consistent with prior research (Cai et al., 2024; Shepherd, Laurens, Matheson, Carr, & Green, 2012), covering key aspects such as patient demographics, clinical characteristics, analytical methods, and the quality of results and conclusions. Each item was assigned a score of 0, 0.5, or 1, indicating the extent to which the criteria were not met, partially met, or fully met, respectively. Of note, the aim of using this checklist was to evaluate the completeness of the information required for our meta-analysis obtained from published studies, not to criticize the investigators or their work. The detailed checklist and the scores for each study are presented in Supplementary Tables S1–S4.

### Neuroimaging meta-analysis

The voxel-wise meta-analysis was performed using Seed-based *d* Mapping with Permutation of Subject Images (SDM-PSI, version 6.22; https://www.sdmproject.com) software, allowing for direct testing of differences between patients with schizophrenia and HCs rather than relying on indirect peak convergence tests. The procedure involved multiple imputation of study images, imputation of subject images, group analyses of subject images for each study and imputation, meta-analyses of study images using the random-effects model for each imputation, and the combination of meta-analysis results using Rubin’s rules (Albajes-Eizagirre, Solanes, Vieta, & Radua, 2019).

In this study, three meta-analyses were conducted, including (1) separate analyses at each illness stage of the disease to identify significant brain activity changes specific to different disease phases in schizophrenia, (2) quantitative comparisons of findings between stages in schizophrenia using a linear model, and (3) a pooled meta-analysis combining all three stages to identify common differences in schizophrenia. The results were corrected using the family-wise error (FWE) correction method based on threshold-free cluster enhancement (TFCE). The statistically significant level was set at FWE-TFCE *p* < 0.05 and a cluster extent of more than 50 voxels. Moreover, to evaluate between-study variability in our findings, Cochran’s *Q* statistic was employed, and the proportion of total variation stemming from heterogeneity was quantified using the *I* ^2^ statistic. Additionally, an assessment of publication bias regarding significant results was conducted through the utilization of Egger’s test.

### Neurosynth cognitive decoding

To explore the cognitive relevance of the spontaneous brain activity changes identified in the neuroimaging meta-analysis, we employed the Neurosynth database (https://neurosynth.org), which provides activation maps for 1307 terms. This analysis allows for the decoding of cognitive processes from brain images by examining spatial correlations between spontaneous brain activity difference maps and the meta-analytic activation map for each term within the database. Following previous studies (Cai et al., 2024; Wang et al., 2025), each unthresholded *z*-map was separated into positive (patients > controls) and negative (patients < controls) maps to facilitate cognitive decoding. This approach enables the identification of potentially distinct cognitive processes associated with hyperactivation and hypoactivation in schizophrenia, thereby enhancing the interpretability of functional alterations. These maps were upload separately for cognitive decoding. The top five cognitive terms (excluding anatomical, demographic, and methodological terms) with the highest absolute correlation coefficients were retained for purposes of interpretation. The earlier process was conducted with brain image findings obtained from three illness stages and pooled group.

### Identification of schizophrenia-associated genes

To identify schizophrenia-associated genes, we used the Hi-C-coupled multimarker analysis of genomic annotation (H-MAGMA) method (https://github.com/thewonlab/H-MAGMA), which integrates chromatin interaction data from Hi-C with GWAS summary statistics to map noncoding variants to their likely target genes. Unlike conventional approaches that map noncoding variants to the nearest gene, H-MAGMA uses three-dimensional chromatin interactions to more accurately link noncoding variants to their regulatory target genes in a tissue-specific manner (Sey et al., 2020). This is particularly useful in psychiatric disorders like schizophrenia, where gene regulation in brain tissues is highly complex (Chen et al., 2018). The ability to map variants to genes based on chromatin interaction data provides a more biologically relevant understanding of how genetic variants affect gene function and contribute to disease pathology.

For this analysis, the largest available GWAS summary statistics were utilized, encompassing data from 53,386 individuals with schizophrenia and 77,258 controls (Trubetskoy et al., 2022). In addition, the H-MAGMA analysis was limited to protein-coding genes, with genes within the major histocompatibility complex region excluded due to its complex LD structure (Sey et al., 2020). The default H-MAGMA settings were employed, and annotation data from both fetal and adult brain tissues were integrated. To control for multiple comparisons, a false discovery rate (FDR) correction was applied separately to gene-level results from each tissue at a significance threshold of *p* < 0.05. Genes that were significantly associated in both tissues were retained for further analysis.

### Transcriptome-neuroimaging association analysis

The brain gene expression data for this study were sourced from the publicly available AHBA database (http://www.brain-map.org), which comprises over 20,000 genes collected from 3702 spatially distinct brain tissue samples, all originating from six human donors without any history of neurological or psychiatric diseases. Details regarding each donor can be found in Supplementary Table S5. Gene expression data preprocessing involved a series of steps using the abagen toolbox (https://www.github.com/netneurolab/abagen): (1) updating probe-to-gene reannotations to eliminate unreliable probe–gene associations; (2) employing intensity-based filtering to remove probes that did not exceed the background noise threshold in at least 50% of samples from all donors; (3) selecting the most differentially stable probes to represent each gene; (4) aligning MNI coordinates of tissue samples with anatomical images using the Advanced Normalization Tools (ANTs; https://github.com/chrisfilo/alleninf); (5) applying scaled robust sigmoid normalization across genes to address potential variations in gene expression driven by measurement errors; (6) conducting scaled robust sigmoid normalization across samples to account for donor-specific differences in gene expression data; and (7) performing normalization to ensure consistency in gene expression patterns across different brain structures, including the cortex, subcortex/brainstem, and cerebellum. Given that our neuroimaging meta-analysis was performed within the default gray matter mask provided by SDM-PSI, we limited our analyses to samples within this mask, resulting in a gene expression matrix of 15,633 genes by 3150 samples. Finally, we intersected the 1968 schizophrenia-associated genes identified through H-MAGMA (as detailed in the Results section) with the gene expression matrix, yielding a final matrix comprising 1613 genes across 3150 samples for subsequent analyses.

We conducted transcriptome-neuroimaging association analysis to identify genes linked to alterations in spontaneous brain activity in schizophrenia. For each illness stage, the mean *z* value within a 6-mm radius sphere centered at the MNI coordinate of each tissue sample was extracted from the unthresholded voxel-wise meta-analysis *z*-map, which represents changes in spontaneous brain activity between individuals with schizophrenia and HCs. Gene-wise Pearson’s correlation analyses were performed across samples to identify genes whose expression levels correlated with schizophrenia-related brain activity changes, with multiple comparisons accounted for using the FDR method (*p* < 0.05). Through these analyses, we identified one distinct gene set for each illness stage, resulting in a total of three gene sets that showed significant spatial correlation with the *z*-maps at the respective stages. By intersecting these three gene sets, we uncovered genes consistently associated with alterations in spontaneous brain activity across all stages of schizophrenia.

Due to the spatial autocorrelation present in both transcriptional and neuroimaging maps, a permutation test consisting of 1000 iterations was conducted to assess whether the number of overlapping genes significantly exceeded what would be expected by random chance. In each iteration, spatial autocorrelation preserving surrogate maps were generated using the Brain Surrogate Maps with Autocorrelated Spatial Heterogeneity (BrainSMASH, https://github.com/murraylab/brainsmash) package (Burt, Helmer, Shinn, Anticevic, & Murray, 2020). The same transcriptome-neuroimaging association procedures were then applied to the surrogate maps, and the number of overlapping genes was recorded to construct a null distribution. The statistical significance of the observed overlap was assessed by comparing the actual count of overlapping genes to the null distribution. The empirical *p* value (*p*_perm_) was determined as the proportion of permutations where the overlapping gene count was equal to or greater than the observed count. The significance threshold was set at *p*_perm_ < 0.05.

### Gene prioritization analysis

To prioritize the genes identified from the transcriptome-neuroimaging association analysis, we conducted a gene prioritization analysis to determine whether the expression levels of these targeted genes were significantly associated with schizophrenia (Liu et al., 2024; Zhao et al., 2025), thereby refining the neuroimaging-based findings. Specifically, we integrated GWAS summary statistics with precomputed expression quantitative trait locus (eQTL) prediction models using Multivariate Adaptive Shrinkage in *R* (MASHR) (Urbut, Wang, Carbonetto, & Stephens, 2018), covering all brain tissues from the Genotype-Tissue Expression (GTEx v8) project (Aguet et al., 2020), including regions such as the amygdala, anterior cingulate cortex, caudate, cerebellar hemisphere, cortex, and hippocampus. Using the S-MultiXcan tool (Plagnol et al., 2019), we combined these eQTL models with GWAS summary statistics from the largest schizophrenia study, evaluating the association between predicted gene expression and schizophrenia. Multiple testing corrections were applied using the FDR method (*p* < 0.05) to ensure robustness. This approach allowed us to prioritize genes with significant expression-level associations and further explore the biological functions of these prioritized genes through gene enrichment analysis.

### Gene enrichment analysis

To investigate the biological functions of the prioritized genes, gene enrichment analysis was conducted using the online tool g:Profiler (https://biit.cs.ut.ee/gprofiler/gost) (Raudvere et al., 2019), incorporating Gene Ontology (GO) biological process terms, Kyoto Encyclopedia of Genes and Genomes (KEGG), and Reactome pathway databases. In addition, gene-category enrichment analysis (GCEA) was used to validate the GO biological process results, accounting for spatial autocorrelation and gene–gene coexpression that may lead to inflated significance (Fulcher, Arnatkeviciute, & Fornito, 2021). In brief, the gene score for each gene was defined as the average of the sum of absolute Pearson’s correlation coefficients obtained during the transcriptome-neuroimaging association analysis in different stages of schizophrenia. Category scores were subsequently calculated by averaging the gene scores within each GO category, and a null distribution for these category-level scores was created by generating an ensemble of 1000 randomized phenotypes while preserving spatial autocorrelation. To assess the statistical significance of the results, the actual category score was compared to this null distribution of category-level scores, allowing for statistical inference on GO categories.

Furthermore, we employed the online tissue-specific expression analysis (TSEA, http://doughertytools.wustl.edu/TSEAtool.html) tool to perform tissue- and temporal-specific expression analyses (Dougherty, Schmidt, Nakajima, & Heintz, 2010), with the aim of determining the specific tissues and developmental stages in which these identified genes were overrepresented. Specific expression analysis across developmental windows leverages human data from the Brainspan atlas (http://www.brainspan.org/). The specificity index probability (pSI) is a statistical measure that quantifies how selectively a gene is expressed in a particular tissue or developmental stage compared to all other reference contexts (Dougherty et al., 2010). In this study, we used a pSI threshold of 0.05 to identify whether the identified gene sets were significantly enriched in specific brain regions or developmental periods. A significance level of FDR-corrected *p* < 0.05 was used for all enrichment analyses.

### Protein–protein interaction (PPI) analysis

Following the gene enrichment analysis, we conducted a PPI analysis to explore the interaction network among the prioritized genes. Using STRING v12.0 (https://cn.string-db.org/), we assessed whether these genes formed a cohesive PPI network. To ensure robustness, only interaction pairs with a confidence score exceeding 0.9 were included in the analysis. The network was further analyzed to identify hub genes – those with the highest degree values, indicating the number of connections or edges associated with each gene. These hub genes are critical nodes within the network and likely play key roles in the molecular mechanisms underlying schizophrenia.

## Results

### Included studies and sample characteristics

**Figure 2.**
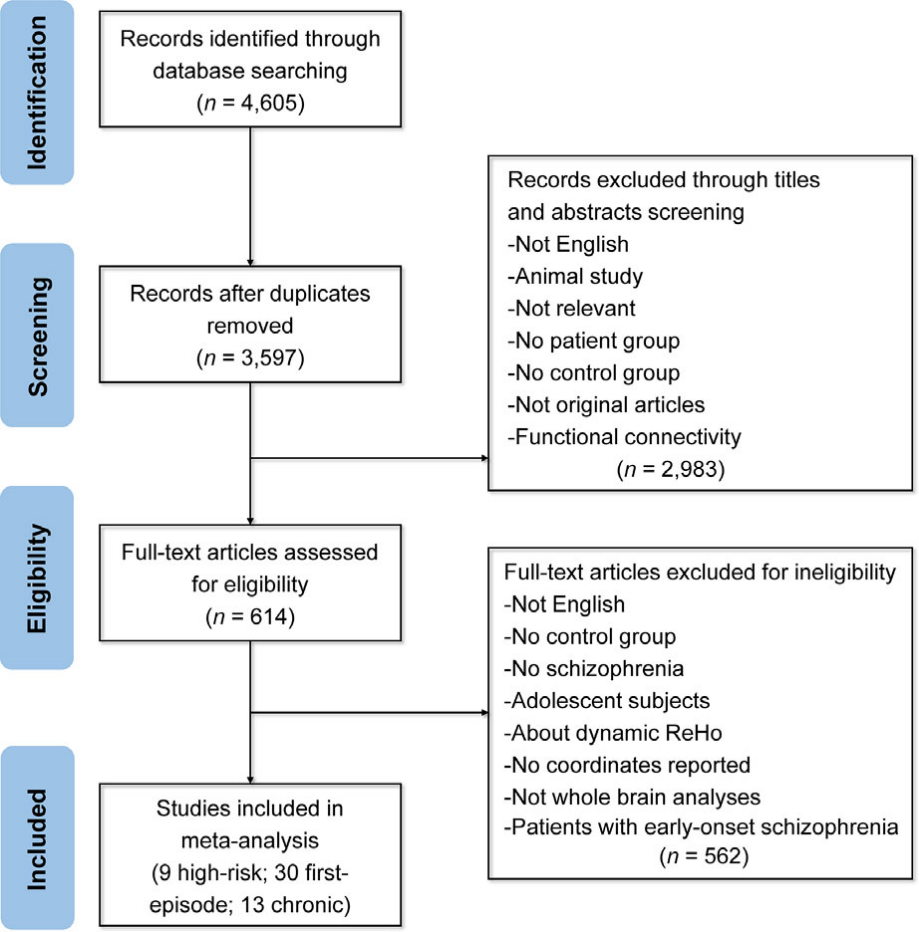
The flowchart of literature search and selection in the meta-analysis. ReHo, ‘regional homogeneity’.

As illustrated in Figure 2, our initial search identified 4605 records, of which 52 studies met inclusion criteria for the meta-analysis. These studies spanned different illness stages: 9 studies of high-risk individuals (409 patients, 475 HCs) contributed 12 datasets; 30 first-episode studies (1842 patients, 1735 HCs) contributed 38 datasets; and 13 chronic-stage studies (1242 patients, 1300 HCs) contributed 22 datasets. In total, 72 datasets across all stages were included in the meta-analysis. Detailed demographic, clinical, and imaging characteristics of all studies are provided in Supplementary Tables S6–S11. Sample-size-weighted *t* tests confirmed no significant age or gender differences between patients and controls in each stage-specific or pooled meta-analysis (all *p*s > 0.05).

### Neuroimaging meta-analysis and cognitive relevance terms

**Figure 3.**
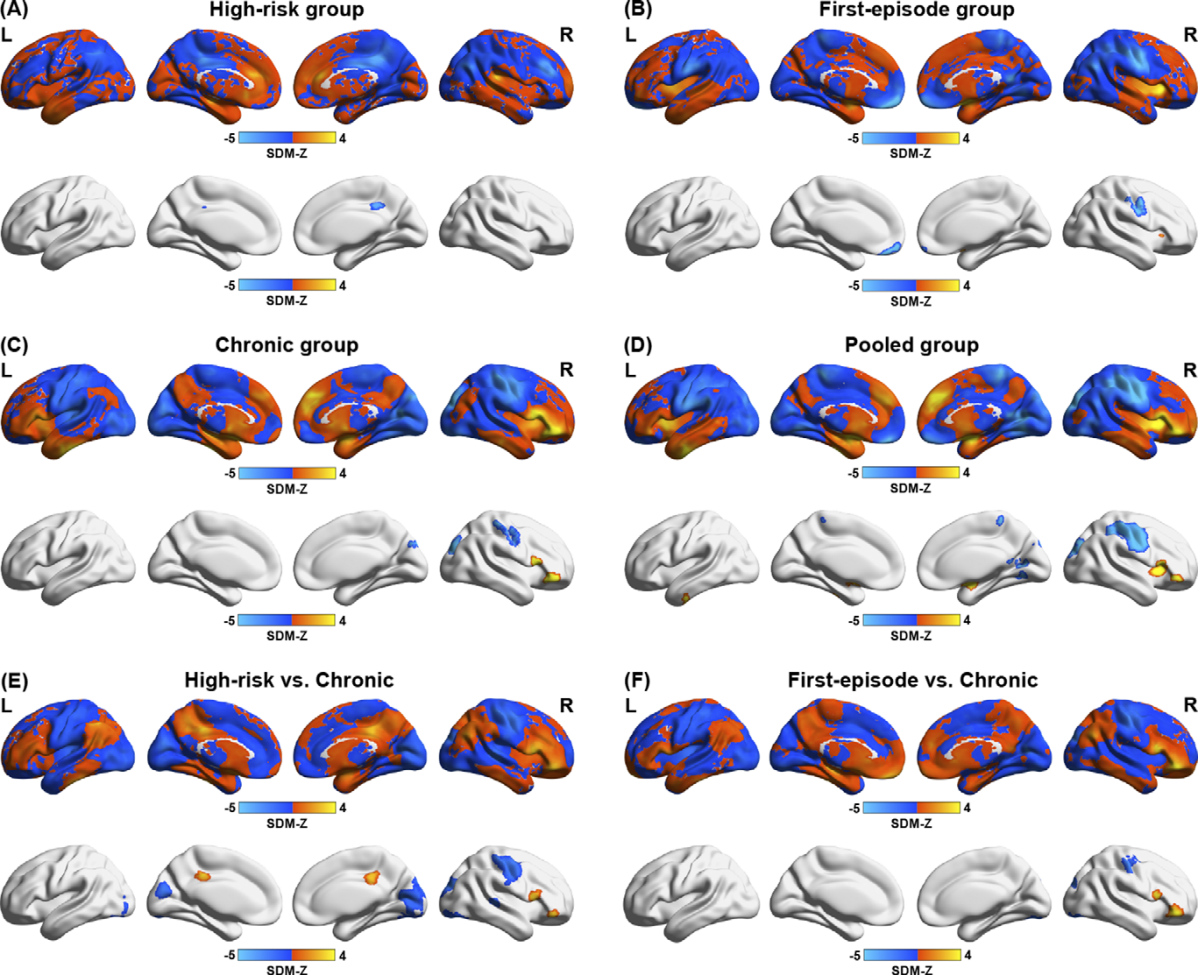
Spontaneous brain activity alterations in patients with schizophrenia. (a) Case–control spontaneous brain activity alterations in the meta-analysis of high-risk stage. (b) Case–control spontaneous brain activity alterations in the meta-analysis of first-episode stage. (c) Case–control spontaneous brain activity alterations in the meta-analysis of chronic stage. (d) The meta-analysis result of the pooled group. (e) Comparison of spontaneous brain activity between the high-risk and chronic groups. (f) Comparison of spontaneous brain activity between first-episode and chronic group. The color bar represents the SDM *z* values, and a positive *z* value indicates increased spontaneous brain activity, while a negative *z* value indicates decreased activity. L, ‘left’; R, ‘right’; SDM, ‘seed-based d mapping’.

In comparison with HCs, individuals at the high-risk stage exhibited decreased spontaneous brain activity in the right median cingulate and paracingulate gyri (Figure 3a). First-episode schizophrenia patients displayed increased activity in the right putamen and decreased activity in the left gyrus rectus and right postcentral gyrus (Figure 3b). For chronic schizophrenia patients, elevated activity was observed in the orbital and opercular parts of the right inferior frontal gyrus, while reduced activity was found in the right postcentral gyrus and superior occipital gyrus (Figure 3c and Supplementary Table S12). Quantitative comparisons revealed significant differences in spontaneous brain activity when comparing the high-risk and chronic stages, as well as the first-episode and chronic stages of schizophrenia. Specifically, in the chronic stage, increased activity was observed in regions of the right inferior frontal gyrus, while prominent reductions were identified in the right postcentral gyrus and superior occipital gyrus compared with other stages. No significant differences were found between the high-risk and first-episode stages. Detailed results are shown in Figure 3e,f and Supplementary Table S14. In the pooled analysis combining all three stages, increased spontaneous brain activity was observed in the orbital part of the right inferior frontal gyrus, left putamen, and left inferior temporal gyrus, while decreased activity was noted in the right postcentral gyrus, superior occipital gyrus, right lingual gyrus, and paracentral lobule (Figure 3d and Supplementary Table S13). Cochran’s *Q* test revealed no between-study heterogeneity for the significant clusters, and Egger’s test indicated no significant publication bias in reported results.

Using Neurosynth cognitive decoding, we identified cognitive terms associated with spontaneous brain activity alterations in schizophrenia patients. Regions with increased spontaneous brain activity were linked to ‘reward’ processes, not only across the three illness-stage groups, but also in the pooled group analysis. Meanwhile, regions with decreased spontaneous brain activity were associated with ‘sensorimotor’, ‘somatosensory’, and ‘primary somatosensory’ functions in both the first-episode and chronic groups. Detailed results are provided in Supplementary Table S15.

### Identification of schizophrenia-related genes and their association with brain activity

Using the H-MAGMA method, we identified 1968 schizophrenia-associated genes. These were intersected with the preprocessed gene expression data from the AHBA database, which retained 15,633 genes after preprocessing. This process resulted in a final set of 1613 genes selected for further analysis. Subsequently, transcriptome-neuroimaging association analyses were conducted to investigate the relationship between gene expression and alterations in spontaneous brain activity across different stages of schizophrenia. These analyses revealed spatial associations between gene expression and brain activity changes in 874 genes for individuals at risk of schizophrenia (Supplementary Table S16), 845 genes for those with first-episode schizophrenia (Supplementary Table S17), and 1048 genes for individuals with chronic schizophrenia (Supplementary Table S18). Across all three illness stages, a total of 441 genes were consistently associated with altered brain activity. The statistical significance of this overlap was confirmed using a spatially constrained permutation test (*p*_perm_ = 0.005, Supplementary Table S19), indicating that the observed overlap was highly unlikely to have occurred by chance. Among these, 199 genes were prioritized through S-MultiXcan, showing significant predicted expression levels associated with schizophrenia. The detailed workflow of gene identification and filtering is presented in Supplementary Figure S1.

### Functional enrichment and PPI analysis

**Figure 4.**
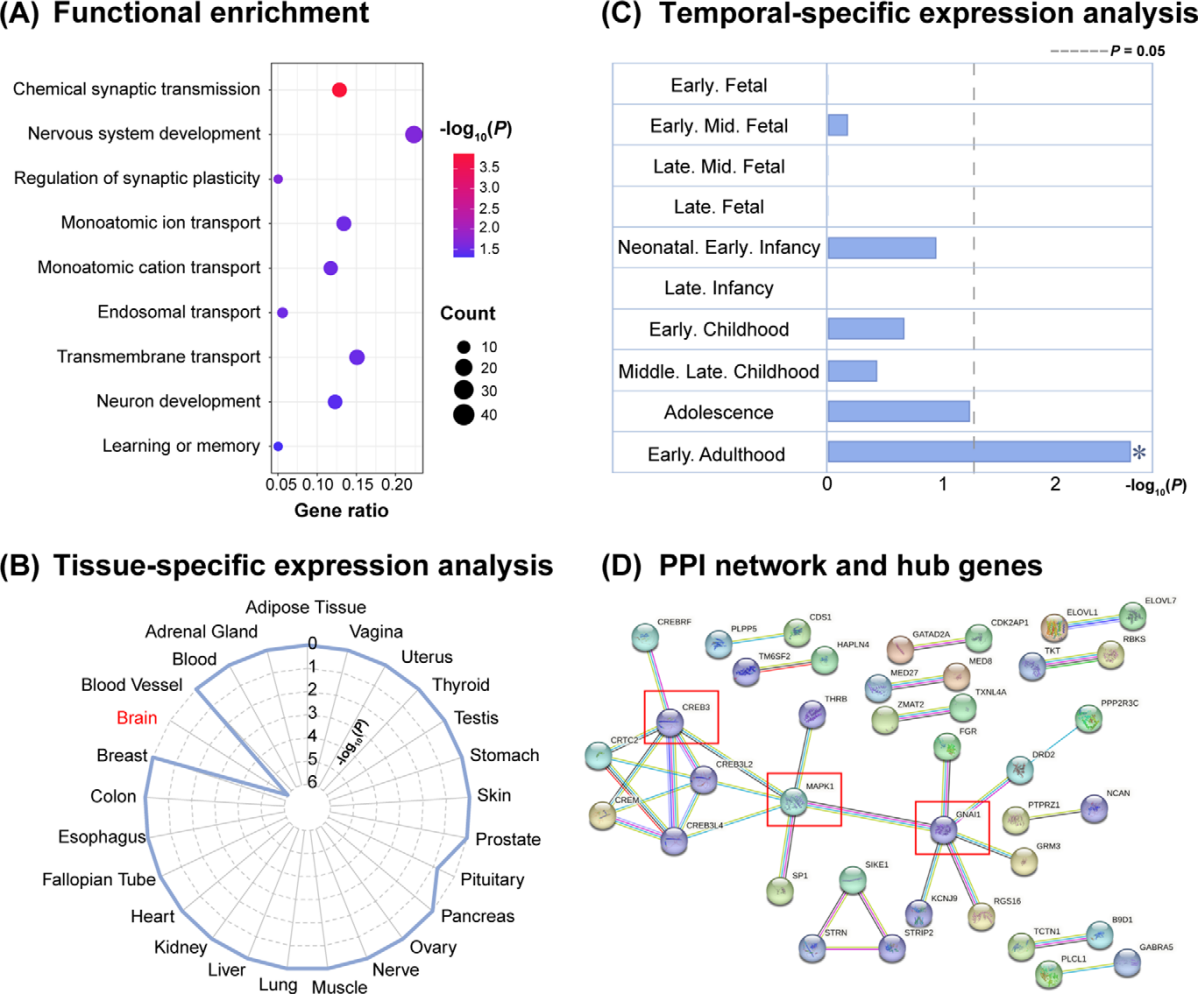
Results of functional enrichment, specific expression, and PPI analyses for genes reliably associated with spontaneous brain activity changes in schizophrenia. (a) Functional enrichment of the identified 199 genes. The *x*-axis denotes gene ratio and the *y*-axis denotes biological process terms. The gene ratio refers to the ratio of the number of intersection size to the number of query size. The size of the bubble represents the intersection number of genes in the term, and the shade of the color represents the significance. (b) Specific expression analyses across tissue types. (c) Specific expression analyses across temporal stages. The *x*-axis shows the temporal stages respectively and the *y*-axis shows the −log_10_(*p*) value, with the gray dashed line indicating the threshold of significance (FDR, *p* = 0.05). Asterisk denotes significance at FDR-corrected *p* < 0.05. (d) The PPI network analysis of the 199 identified genes, with red boxes representing hub genes. FDR, ‘false discovery rate’; PPI, ‘protein–protein interaction’.

Functional enrichment analysis revealed that the 199 genes associated with spontaneous brain activity alterations in schizophrenia were significantly involved in biological processes such as nervous system development, chemical synaptic transmission, and regulation of synaptic plasticity (Figure 4a and Supplementary Table S20). All reported GO biological process terms were further confirmed using the GCEA approach. Additionally, one significant pathway was identified in the Reactome database, while no significant enrichment was found in KEGG. Tissue-specific expression analysis demonstrated that these genes were specifically expressed in brain tissue (Figure 4b), while temporal-specific expression analysis indicated a preferential expression during the young adulthood developmental stage, with extensive involvement in the cortex (Figure 4c). In addition, PPI network analysis for the 199 genes identified a network of 204 interconnected proteins with 35 edges, which was significantly higher than expected (*P* = 8.74 × 10^−6^). Among these proteins, *CREB3*, *GNAI1*, and *MAPK1*, with the highest degree values, were identified as hub genes, suggesting their central roles in the underlying molecular mechanisms of schizophrenia (Figure 4d).

## Discussion

To our knowledge, this study represents the first effort to explore the genetic and neurobiological mechanisms underlying brain activity alterations across different stages of schizophrenia. Neuroimaging meta-analyses revealed both common and distinct spontaneous brain activity patterns associated with high-risk, first-episode, and chronic schizophrenia. Specifically, stage-specific alterations were observed in regions such as the right median cingulate gyrus, right putamen, and right inferior frontal gyrus, which varied in activity across the stages. By utilizing H-MAGMA method, transcriptome-neuroimaging association analysis and gene prioritization analysis, 199 genes were prioritized to be consistently linked to these brain activity changes, which enriched in key biological processes, including synaptic transmission and nervous system development. These findings not only provide a comprehensive view of the progression of brain activity changes in schizophrenia, but also offer valuable insights into the genetic pathways that could be targeted for stage-specific interventions.

Individuals at high-risk stage showed significantly decreased spontaneous brain activity in the right median cingulate/paracingulate gyri compared with HCs. This region is an important part of the limbic system, which is responsible for regulating emotional disorders, and aberrant functioning of these brain regions is linked to negative emotions (Riemann et al., 2010). Consistent with this, recent evidence suggests that altered functional connectivity within large-scale brain networks involved in emotional and self-referential processing, such as the default-mode network, may contribute to vulnerability to stress-related psychopathology, with psychological resilience playing a mediating role (Liu et al., 2023). Furthermore, disruptions in structure–function coupling have also been linked to mood-related symptoms in other clinical populations, such as older women with subthreshold depression (Suo, Chen, Kemp, Wu, & Wang, 2025), suggesting potentially shared neurobiological mechanisms across psychiatric conditions. Emotion impairment has long been considered one of the prominent, core features of schizophrenia (Tremeau, 2006). Previous study indicated that dysfunction in the limbic system is associated with the onset of schizophrenia, particularly in high-risk group, exhibiting specific neural activity patterns in executive function and emotional processing (Hart et al., 2013). Therefore, alterations of the right median cingulate/paracingulate gyri in limbic system may serve as potential vulnerability markers for individuals at high-risk stage of schizophrenia.

Our results showed increased spontaneous brain activity in the right putamen and decreased in the left gyrus rectus in patients with first-episode schizophrenia. The putamen is a part of the striatum, which holds a crucial role in cognitive processing and is an integral component of various neuroanatomical linked to emotional processing and regulation (Cheesman, 2005; Lindquist, Wager, Kober, Bliss-Moreau, & Barrett, 2012). Previous investigations focusing on functional and metabolic aspects have revealed heightened dopamine neurotransmission and increased activity within the striatum among individuals diagnosed with schizophrenia (McCutcheon, Krystal, & Howes, 2020; Meyer-Lindenberg et al., 2002). Previous functional evidence also suggested that elevated ALFF and ReHo in striatal regions in patients with schizophrenia (Li et al., 2023; Zhang et al., 2023), which is consistent with our meta-analysis results. The role of the striatum in schizophrenia is further substantiated by the dopamine hypothesis of schizophrenia, which suggests the presence of hyperdopaminergic within the striatum (Howes & Kapur, 2009; McCutcheon, Abi-Dargham, & Howes, 2019; Schmitt et al., 2009). It is proposed that the transient accumulation of excessive spontaneous dopamine can interact with the striatal signaling pathway through stimulation, thereby attributing significance to irrelevant external or internal stimuli for a temporary period (Kapur, 2003; Maia & Frank, 2017). Therefore, it can be inferred that the alterations in the striatum may associate with the delusion symptom of patients with schizophrenia (McCutcheon et al., 2019). The gyrus rectus is a core part of frontal lobe, which is associated with executive functions including advanced cognitive abilities such as working memory, inhibitory control, cognitive flexibility, planning reasoning, and problem solving (Friedman & Robbins, 2021; Smith & Jonides, 1999; Stuss & Levine, 2002). The executive dysfunction is a core feature of schizophrenia resultant of neural mechanisms, which are related to its etiology and onset (Brown et al., 2009; Hutton et al., 2004). Additionally, it is thought that the extent of executive dysfunction is linked to the prognosis and functional responses of the individuals (Eisenberg & Berman, 2010). Decreased spontaneous brain activity in the frontal lobe may be linked to the impairment of executive function observed in patients with first-episode schizophrenia.

Individuals with chronic schizophrenia exhibited increased spontaneous brain activity in the orbital part and opercular part of right inferior frontal gyrus and decreased in the superior occipital gyrus relative to HCs. The inferior frontal cortex plays a crucial role in affect modulation and empathy, both of which are impaired in schizophrenia (Leigh et al., 2013; Zhao, Zhang, et al., 2022). The right superior occipital gyrus, positioned within the occipital lobe, serves as a crucial component of the visual cortex, which is responsible for receiving, segmenting, and integrating visual information (Leopold, 2012; Wandell, Dumoulin, & Brewer, 2007). Impairment in this area is consequently linked to visual perception disruptions in individuals with schizophrenia, including visual hallucinations and distortions (Butler, Silverstein, & Dakin, 2008). Previous studies have investigated significantly lower ReHo values in the visual cortex and visual cognition impairment and visual processing deficits in schizophrenia, which is in accordance with our findings (Butler et al., 2001; Huang et al., 2022). These findings highlight that alterations in the frontal and occipital regions may be key to understanding the persistent emotional and sensory deficits in chronic schizophrenia.

When comparing changes in brain regions across the high-risk, first-episode, and chronic stages of schizophrenia, the trends appear to vary across different brain regions rather than showing a uniform progressive alteration. Certain regions, such as the medial and lateral prefrontal cortex, insula, and anterior temporal lobe, exhibit more evident progressive changes, while posterior regions, including parts of the parietal and occipital lobes, do not follow a clear progressive pattern. Notably, the high-risk stage demonstrates significant differences compared to the other two stages, which might reflect distinct early pathological processes. Some of the regional inconsistencies may stem from methodological heterogeneity across the included studies, including variability in scanner models, imaging protocols, and preprocessing procedures, which may impact the robustness and comparability of meta-analytic findings. Furthermore, the relatively small sample size of high-risk group could influence the observed group-level differences in some brain regions, which may affect the robustness of the findings. Additionally, the relationship between spontaneous brain activity at the high-risk stage and later disease progression may be nonlinear, involving complex or compensatory mechanisms. Supporting this view, structural MRI studies have demonstrated that abnormalities in regions such as the prefrontal and cingulate cortices can evolve over time and contribute to symptom development and treatment resistance (Paul et al., 2024). Despite the lack of significant differences between the high-risk and first-episode stages, the observed differences between the high-risk and chronic stages, as well as between the first-episode and chronic stages, highlight noticeable changes in spontaneous brain activity as the disease progresses. These findings suggest distinct pathological mechanisms between early and late stages of schizophrenia, with the chronic stage showing more concentrated abnormal activity. This supports the hypothesis of functional reorganization during disease progression, where the brain adapts by redistributing resources under increasing pathological stress (Faget-Agius et al., 2013). Interestingly, the right hemisphere remains particularly involved in spontaneous brain activity across all stages, with key regions such as the right inferior frontal gyrus and right postcentral gyrus exhibiting notable changes. This suggests that alterations in right hemisphere regions associated with emotional regulation, motor control, and sensory processing may play a critical role in the pathophysiology of schizophrenia (Larabi, van der Meer, Pijnenborg, Curcic-Blake, & Aleman, 2018; Tu et al., 2010).

Based on the neuroimaging findings, our transcriptome-neuroimaging association analysis identified 199 genes consistently linked to brain activity alterations across all stages of schizophrenia. The prioritization of these genes through S-MultiXcan emphasizes their potential role in the neurobiological processes driving the disorder, particularly those related to nervous system development, synaptic transmission, and synaptic plasticity. The process of nervous system development involves crucial steps such as neurogenesis, migration, synapse formation, and neural circuit establishment (Allen & Lyons, 2018; Silbereis, Pochareddy, Zhu, Li, & Sestan, 2016; Sousa, Meyer, Santpere, Gulden, & Sestan, 2017). Abnormal development of the nervous system can lead to disruptions in neural circuits, affecting neural transmission and regulation, thereby resulting in the manifestation of schizophrenia symptoms (Insel, 2010; Lewis & Levitt, 2002). Chemical synaptic transmission involves the release, reception, and modulation of neurotransmitters at synapses (Alger, 2002; Veletić, Mesiti, Floor, & Balasingham, 2015). In schizophrenia, disruptions in dopamine, glutamate, and gamma-aminobutyric acid (GABA) systems have been widely implicated (McCutcheon, Krystal et al., 2020; Rowland et al., 2013; Yee et al., 2005). Altered dopamine signaling is linked to positive symptoms (Abi-Dargham, 2004; Lyon et al., 2011), while glutamatergic and GABAergic dysfunctions contribute to cognitive deficits and impaired inhibitory control, respectively (Gonzalez-Burgos, Fish, & Lewis, 2011; Tsai & Coyle, 2002). These disruptions likely play a key role in the abnormal resting state activity seen in schizophrenia. Synaptic plasticity, essential for learning and memory (Takeuchi, Duszkiewicz, & Morris, 2014; Whitlock, Heynen, Shuler, & Bear, 2006), is disrupted in schizophrenia, leading to abnormal connectivity and impaired information processing (Daskalakis, Christensen, Fitzgerald, & Chen, 2008; Savanthrapadian et al., 2013). Alterations in synaptic proteins, such as NMDA receptors, may hinder neural circuits’ adaptability, contributing to the brain activity changes observed in schizophrenia (Jackson, Homayoun, & Moghaddam, 2004). Furthermore, the identification of hub genes, such as *CREB3*, *GNAI1*, and *MAPK1*, in the PPI network further highlight the importance of these genes in the molecular pathways associated with schizophrenia. These findings not only advance our understanding of the genetic underpinnings of schizophrenia, but also provide potential targets for therapeutic interventions aimed at modifying these key biological processes.

There are several limitations to acknowledge in this study. First, the number of studies included in the meta-analysis varied across different stages of schizophrenia, resulting in inconsistent statistical power, which may impact the comparability of findings across stages. Second, the reliance on cross-sectional neuroimaging data limits our ability to track the dynamic progression of brain activity changes over time in individuals with schizophrenia. Longitudinal studies would be needed to gain a clearer understanding of how these alterations evolve across different stages of the disorder. Third, while coordinate-based meta-analysis is well established, its reliance on reported peak coordinates rather than full statistical maps leads to lower spatial precision compared to image-based approaches, potentially affecting the anatomical accuracy of the findings. Fourth, the transcriptome-neuroimaging spatial association analysis did not use data from the same individuals for both gene expression and neuroimaging. While gene expression data were derived from postmortem brains in the AHBA database, the neuroimaging data came from living individuals with schizophrenia. This discrepancy may result in the exclusion of genes with significant individual-level expression variability in the transcriptome-neuroimaging association. Finally, only the GO enrichment analysis results were validated using ensemble-based null models due to the limitations of the toolbox used, which only supports GCEA with GO biological process terms.

## Conclusion

In conclusion, we conducted a neuroimaging meta-analysis and transcriptome-neuroimaging association analysis to investigate spontaneous brain activity alterations across different stages of schizophrenia. We identified stage-specific changes in brain regions, including the right median cingulate gyrus, right putamen, and right inferior frontal gyrus. Additionally, 199 genes were prioritized to be consistently associated with these brain activity changes. Functional enrichment analysis revealed key biological processes, such as synaptic transmission and nervous system development, that may be involved in the progression of schizophrenia. These findings contribute to a deeper understanding of the disorder’s biological underpinnings and may inform the development of more effective diagnostic tools and therapeutic interventions.

## Supporting information

Zhang et al. supplementary material 1 Zhang et al. supplementary material

Zhang et al. supplementary material 2 Zhang et al. supplementary material

## Supplementary material

The supplementary material for this article can be found at http://doi.org/10.1017/S0033291725102006.
