# Supplementary material for: From risk to chronicity: genetic and neuroimaging insights into the evolving patterns of spontaneous brain activity in schizophrenia
Source: Psychol Med. 2025 Oct 10;55:e306. doi: 10.1017/S0033291725102006 (PMC12551581; doi:10.1017/S0033291725102006)

**Figure S1.** **Workflow of gene identification in the transcriptome-neuroimaging association analysis.** A total of 1,968 schizophrenia-related genes were intersected with 15,633 genes retained after preprocessing the AHBA expression data, yielding 1,613 risk genes. These risk genes were then used in transcriptome-neuroimaging association analyses for the high-risk, first-episode, and chronic stages of schizophrenia, resulting in 874, 845, and 1,048 associated genes, respectively. A total of 441 genes overlapped across all three stages and were subjected to gene prioritization analysis, yielding 199 candidate genes potentially involved in disease progression.

Abbreviations: AHBA, Allen Human Brain Atlas.


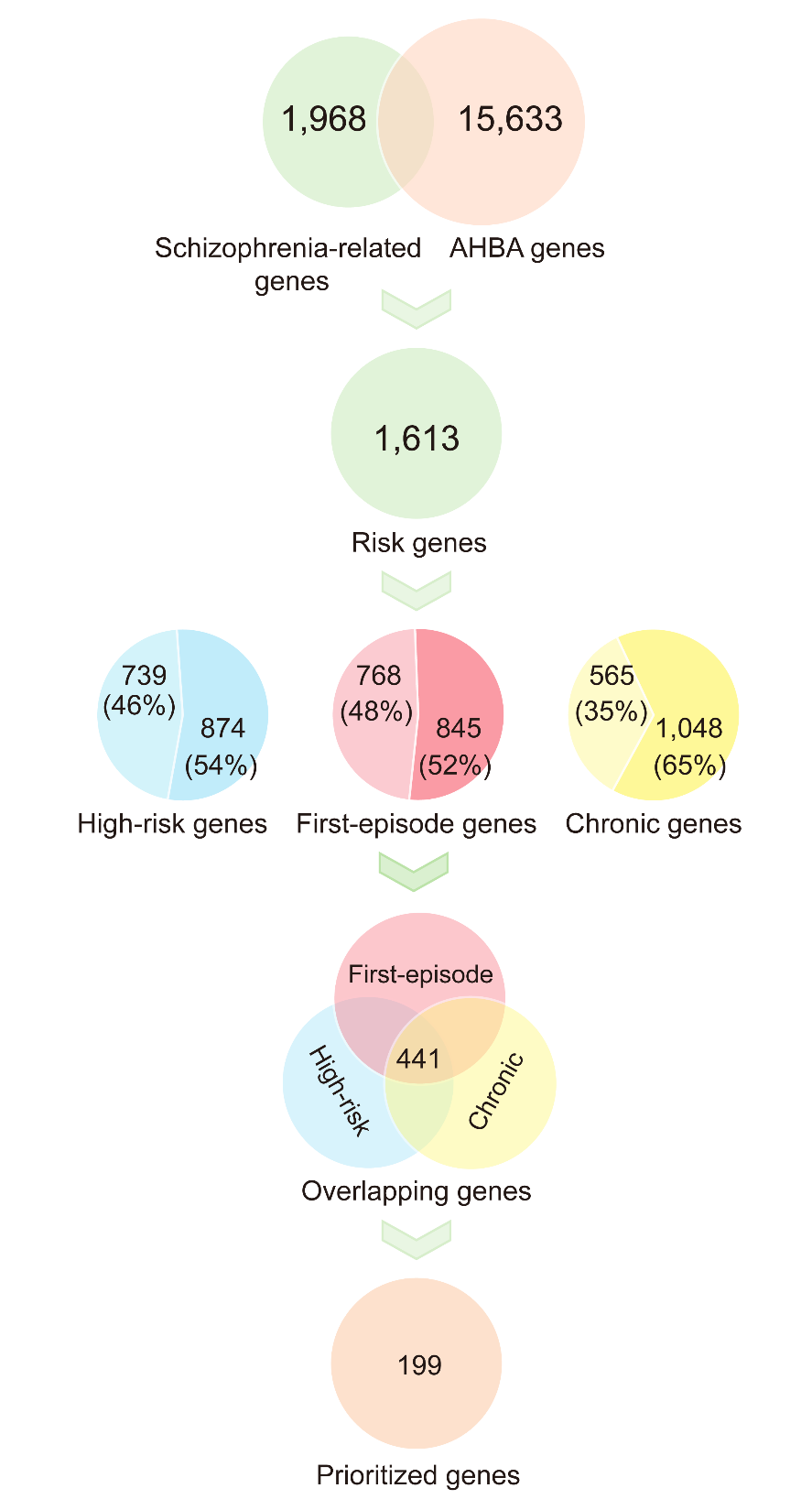

Supplement: Zhang et al. supplementary material 2 — Zhang et al. supplementary material [file S0033291725102006sup002.docx]
